# Derivation and validation of 10-year all-cause and cardiovascular disease mortality prediction model for middle-aged and elderly community-dwelling adults in Taiwan

**DOI:** 10.1371/journal.pone.0239063

**Published:** 2020-09-14

**Authors:** Tsai-Chung Li, Chia-Ing Li, Chiu-Shong Liu, Wen-Yuan Lin, Chih-Hsueh Lin, Shing-Yu Yang, Cheng-Chieh Lin

**Affiliations:** 1 Department of Public Health, College of Public Health, China Medical University, Taichung, Taiwan; 2 Department of Healthcare Administration, College of Medical and Health Science, Asia University, Taichung, Taiwan; 3 School of Medicine, College of Medicine, China Medical University, Taichung, Taiwan; 4 Department of Medical Research, China Medical University Hospital, Taichung, Taiwan; 5 Department of Family Medicine, China Medical University Hospital, Taichung, Taiwan; Karolinska Institutet, SWEDEN

## Abstract

Prediction model mainly focused on specific diseases, such as diabetes, hypertension, cardiovascular disease, or patients with cancer, or populations of Europe and America, thereby limiting its generalization. This study aimed to develop and validate a 10-year mortality risk score by using data from a population-representative sample of adults. Data were collected from 2,221 Taichung Community Health study participants aged ≥40 years. The baseline period of the study was 2004, and all participants were followed up until death or in 2016. Cox’s proportional hazards regression analyses were used to develop the prediction model. A total of 262 deaths were ascertained during the 10-year follow-up. The all-cause mortality prediction model calculated the significant risk factors, namely, age, sex, marital status, physical activity, tobacco use, estimated glomerular filtration rate, and albumin-to-creatinine ratio, among the baseline risk factors. The expanded cardiovascular disease (CVD) mortality prediction model consisted of six variables: age, sex, body mass index, heart disease plus heart disease medication use, stroke plus medication use, and ankle–brachial index. The areas under receiver operating curves of the 3-, 5- and 10-year predictive models varied between 0.97, 0.96, and 0.88 for all-cause mortality, and between 0.97, 0.98, and 0.84 for expanded CVD mortality. These mortality prediction models are valid and can be used as tools to identify the increased risk for mortality.

## Introduction

During the past two decades, public health and medical care have been improved to reduce infant mortality and low-age mortality [1]. The mortality risk of middle-aged and elderly adults of the general population is greater than that of young adults, especially due to cancers, cerebrovascular disease (CVA), diabetes, cardiovascular heart disease (CHD), and kidney disease [2]. Among these chronic diseases, CHD, CVA, diabetes, and kidney disease are considered as expanded cardiovascular diseases and are the primary causes of death worldwide [3]. CHD, CVA, diabetes, and kidney disease were the 2nd, 4th, 5th, and 9th leading causes of death in 2017, respectively, and accounted for more than 27% of total deaths in Taiwan [2].

Similar to many countries, Taiwan has progressively increased number of people aged 40 or above [4]. When the age structures of populations become less youthful, a tool to predict mortality risk for middle-aged and elderly adults becomes increasingly important for research, policy, and clinical practice. In addition, this rapid demographic change increases the costs on medical care and health systems [5]. We should focus on patients who would benefit the most from the preventive and therapeutic services to decrease costs and improve the efficiency. Prediction model shows the potential to support the evaluations of health economics for the allocation of healthcare resources and to provide insights into public health policy.

An easy-to-use risk-point system may be suitable in clinical practice and could easily calculate individual own mortality risk and monitor this risk over time. Mortality risk score can be applied broadly to help people and their primary physicians in making informed decisions by using the remaining life expectancy estimations [6–8]. Applying these risk-point systems provides better risk or prognostic reclassification value than clinical risk factors alone. However, most risk score systems for predicting overall mortality have been developed to focus in Western general population [9–17], and few studies in non-Western general population [18–21]. Three works explored the 5-year mortality risk by focusing on middle-aged or elderly adults residing in a community in Taiwan [18–20]. Two of these studies considered frailty markers [19, 20], and one considered cardiovascular/metabolic, inflammatory, and neuroendocrine markers [18]. None of them considered arterial stiffness, developed a prediction model for expanded cardiovascular mortality, and/or adopted the derived risk-score system. Two noninvasive measures of arterial stiffness, brachial–ankle pulse wave velocity (baPWV) and ankle–brachial index (ABI), were used frequently in community studies [22, 23]. The baPWV, reflecting arterial stiffness, arteriosclerosis, and atherosclerosis, is a marker of the severity of vascular damage [22]. ABI test is a quick, noninvasive way to examine the risk of peripheral artery disease (PAD), a chronic condition with narrowed or blocked arteries in the legs or arms. Arterial stiffness is an important predictor of mortality [24], and people with PAD are associated with an increased risk of CHD. Therefore, the present study aimed to develop and validate a risk-point system to predict long-term all-cause and expanded cardiovascular mortality in a community setting. In addition to traditional risk factors, arterial stiffness is included in this system.

## Materials and methods

### Study design and subjects

The Taichung Community Health study (TCHS), a community-based prospective cohort study, has been conducted among 2359 residents aged 40 years and above in Taichung City, Taiwan in October 2004. The sampling approach was a two-stage sampling design with a sampling rate proportional to the size within each stage. The sampling units for the first and second stages were Li (blocks of household units) and individual. The first stage had a selection probability of 0.125 for eight city districts, and 39 Lis were selected. Finally, 4280 individuals were selected at the second stage. We found 750 ineligible persons who were excluded at household visits and the number of study subjects for the listed reasons of ineligibility was shown in S1 Table. A total of 2359 individuals participated, and the overall response rate was 66.83%. All participants of the TCHS were invited to partake in the study by letter and phone and to provide fasting blood sample. Data were collected using a standardized questionnaire via face-to-face interview and health checkup. By linking the National Registry of Death dataset, we obtained information on the individual’s death or survival and survival times. We further excluded 138 individuals with missing values of baseline variables. A total of 2,221 participants were randomly allocated into the derivation and validation sets in a 2:1 ratio. This study was conducted after obtaining approval from the Institutional Review Board of China Medical University Hospital (DMR92-IRB-144, DMR93-IRB-138 & CMUH106-REC2-143) and all methods were performed in accordance with the relevant guidelines and regulations. Each participant provided his/her written informed consent.

### Measurements

#### Questionnaire

A standardized questionnaire providing sociodemographic characteristics, lifestyle behaviors, history of diseases, and medication use was utilized to collect data. The questionnaire included items about marital status, educational attainment level, income level, personal history of established hypertension, the presence of diabetes, heart diseases, stroke, hyperlipidemia, gout and cancer, current medications of anti-diabetes, hypertension, heart diseases, stroke and hyperlipidemia, smoking habits, alcohol intake, and leisure-time physical activity.

#### Anthropometric measurement

The anthropometric measurements consisted of body weight, height, body mass index (BMI), and blood pressure measurements. We measured weight in kilograms to the nearest 0.5 kg with an electronic medical scale (SECA, Hamburg, Germany) and height in meters to the nearest 0.5 cm with a fixed stadiometer (SECA). We derived BMI as weight in kilograms divided by the square of height in meters (kg/m^2^). We classified persons with BMI ≥25 kg/m^2^ as overweight and BMI ≥30 kg/m^2^ as obese. An electronic device in a seated position (Colin, VP-1000, Japan) was used to measure the blood pressure.

#### Laboratory examination

Following a 12 h overnight fast, a blood sample was drawn from an antecubital vein in the morning and was sent for analysis within 4 h. Blood was tested by a biochemical autoanalyzer (Beckman Coluter, Lx-20, USA) with focus on low-density lipoprotein-cholesterol, high-density lipoprotein-cholesterol, triglyceride, uric acid, urine albumin, and creatinine at the Clinical Laboratory Department of China Medical University Hospital. We measured the serum insulin level by a commercial ELISA kit (Diagnostic Products Corp., Los Angeles, CA, USA) and the fasting plasma glucose level by a glucose oxidase method (Astra-8, Beckman, CA, USA). The inter-assay and intra-assay coefficients of variation (CV) for insulin were 8.7% and 3.4%, respectively. We estimated insulin sensitivity by a homeostasis model assessment (HOMA-IR) equation. The inter-assay precision CV of urinary creatinine (Jaffe’s kinetic method) and albumin (colorimetyl BCP) were both <3.0%. The lowest detection limits of urinary creatinine and albumin were <884 μmol/L and <10 g/L, respectively. We measured high sensitive C-reactive protein (hs-CRP) by a latex particle-enhanced immunoassay (TBA-200FR, Tokyo, Japan) with the inter-assay and intra-assay CVs of <2.0% and 1.9%, respectively, and a low detection limit of the assay of 0.1 mg/L.

#### Pulse wave velocity and ABI

baPWV and ABI were measured using pressure cuffs wrapped around the participant’s brachium and ankle [25]. The results of validation studies by using Pearson’s correlation coefficients and Bland–Altman plots revealed that this device has good validity and reproducibility [25–27], and the automatic non-invasive device could be a good screening tool for subclinical vascular pathology [28]. We obtained baPWV and ABI measurements using an automatic volume-plethysmographic device from PWV/ABI (PWV/ABI; Colin Co. Ltd., Komaki, Japan) while simultaneously recording the blood pressure, PWV, an electrocardiogram, and hear sounds [25, 29]. The device reproducibility has been documented (CV = 8.4% and reproducibility coefficient = 0.98) [25]. The subjects were examined in the supine position after at least 5 min of rest. Pneumatic cuffs were placed on both the brachia and ankles with a sensor that analyzes the volume pulse form. An oscillometric pressure sensor was also used to measure the blood pressure. The electrodes of the electrocardiograph were placed on both wrists, and the cuffs were connected. The baPWV was calculated from the maximum of right PWV (right upper arm to right ankle) and left PWV (left upper arm to left ankle). High values of baPWV indicate high arteriosclerosis severity. The high baPWV value was selected as the representative baPWV for indexing atherosclerosis. ABI was derived by dividing the systolic blood pressure at the arteries near the ankle by the systolic blood pressure in the arms. Low ABI values indicate great arteriosclerosis severity. The representative ABI value for indexing atherosclerosis was examined by the low value between the right and left ABIs.

### Outcome ascertainment

The primary outcome measure was all-cause and expanded cardiovascular death status, derived from record linkage with cause of death data in the Health and Welfare Data Science Center database. The time of follow-up began at entry date (index date) and ended up at the time of death, or on December 31, 2016. Expanded cardiovascular disease mortality was defined as cardiovascular disease (ICD-9-CM codes 390–459, ICD-10-CM codes I00–I99) plus diabetes (ICD-9-CM code 250, ICD-10-CM codes E10–E14) plus kidney diseases (ICD-9-CM 580–589; ICD-10-CM N00–N29) [3].

### Statistical analysis

The participants’ baseline characteristics were presented as frequency (percentage) and means±standard deviations (SDs) when appropriate. We calculated the standardized effect sizes to assess the comparability of the baseline characteristics between the derivation and validation sets. Standardized effect sizes less than 0.1 indicated that the differences between the derivation and validation sets were trivial [30]. To build the prediction models, we used the multivariate Cox’s proportional hazards models. The variables with P-values from age adjustment analysis less than 0.05 were entered in multivariate analysis simultaneously. And then backward elimination approach was used to delete those variables with P-values >0.05 one by one. Only variables with P<0.05 could be retained in the final multivariate model. The proportionality of hazard assumption was confirmed in the final model by examining a bunch of the product terms of each independent variable with log follow-up time. This proportionality assumption was held for both all-cause and expanded CVD mortality (P-values: 0.2556 and 0.2710, respectively). We developed the 10-year mortality prediction models by using the derivation set and assessed the models’ predictive accuracy via the validation set. The survival curves were estimated by Kaplan–Meier method. The steps in the development of the predictive models are based on the Framingham heart study [31]. Mortality probabilities were estimated by the equation: $\hat{p}=1-S_{0}{\left(t\right)}^{exp(\sum \beta_{i}\times X_{i}-\beta_{i}\times {\bar{X}}_{i})}$, where *S*_0_(*t*) is the baseline disease-free probability; *β_i_* is the regression coefficient for *X*_*i*_; and ${\bar{X}}_{i}$ is the mean level of *X*_*i*_. Discriminatory power was assessed by the receiver operating characteristic (ROC) curve analysis. For considering the effect of individuals who are alive may die later due to longer study follow-up, the estimate of area under curve by time-dependent ROC curve analysis was also reported. Hosmer–Lemeshow *x*^2^ tests were used to evaluate the goodness-of-fit by comparing the observed and predicted events of death. We performed 5000 times bootstrap resampling to assess the internal validation for the potential for overfitting or “optimism” [32]. We also assessed the model calibration for the agreement between the model-predicted and observed probabilities. We calculated the intercept to evaluate whether or not the predictions are systematically too low or too high. Intercept value close to zero implies no systematic deviation in estimation of predicted probabilities. Moreover, calibration slope was estimated for extremeness of the predicted probabilities. Slope value close to one indicated no overfitting or “optimism” of a model. Statistical analysis was performed in SAS version 9.4 (SAS Institute Inc., Cary, NC). All statistical significance tests used two-tailed with P-value <0.05.

## Results

A total of 262 deaths occurred during follow-up over 10 years, and more than 29% showed expanded CVD deaths. The event rates for death and expanded CVD death as per 1000 person years were 6.04 to 7.12, 9.84, and 1.81, 1.92, 2.95 in the 3-, 5-, and 10-year periods, respectively. Distributions of the baseline characteristics in the derivation and validation sets were generally similar as shown in Table 1. Mean age was 56 years (SD of 11 years) in both sets, and the number of men was approximately 50% in the derivation and validation sets. All standardized differences of less than 0.1 indicated negligible differences between the derivation and validation sets [33].

**Table 1:** Baseline characteristics of the study subjects in the derivation and validation sets.

Variables	Derivation set (n = 1,480)		Standardized effect size
	MEAN±SD† or n (%)	MEAN±SD† or n (%)	
***Socio-demographic factors***			
Age (years)	56.70±11.79	56.48±11.09	0.02
Sex			
Male	741 (50.07)	352 (47.50)	0.05
Female	739 (49.93)	389 (52.50)	-0.05
Marital status			
Single, widow	246 (16.62)	124 (16.73)	0.00
Married	1234 (83.38)	617 (83.27)	0.00
Educational attainment level			
≤6 years	340 (22.97)	184 (24.83)	-0.04
7–12 years	621 (41.96)	291 (39.27)	0.05
≥13 years	519 (35.07)	266 (35.90)	-0.02
Income level (NT$)			
≤20,000	320 (21.62)	145 (19.57)	0.05
20,001–40,000	386 (26.08)	205 (27.67)	-0.04
40,001–70,000	424 (28.65)	198 (26.72)	0.04
>70,000	350 (23.65)	193 (26.05)	-0.06
Waist circumference (cm)†	81.35±10.11	81.27±9.88	0.01
Body mass index (kg/m^2^)†	24.29±3.36	24.30±3.24	0.00
***Lifestyle behaviors***			
Smoking	241 (16.28)	115 (15.52)	0.02
Alcohol drinking	357 (24.12)	161 (21.73)	0.06
Physical activity	1007 (68.04)	489 (65.99)	0.04
***Biomarker***†			
Systolic blood pressure (mm Hg)	135.55±21.84	135.19±22.08	0.02
Diastolic blood pressure (mm Hg)	78.9±12.53	78.92±12.57	0.00
Fasting plasma glucose (mg/dL)	102.58±27.96	104.84±29.71	-0.08
Insulin (uIU/ml)	8.55±6.97	8.27±6.26	0.04
Total cholesterol (mg/dL)	202.44±36.92	204.15±37.56	-0.05
Triglyceride (mg/dL)	120.15±98.02	122.94±88.55	-0.03
High-density lipoprotein (mg/dL)	46.22±12.61	45.39±12.61	0.07
Low-density lipoprotein (mg/dL)	126.85±33.33	128.3±33.43	-0.04
hsCRP (mg/dL)	0.25±0.59	0.22±0.28	0.06
Creatinine (mg/dL)	0.91±0.32	0.89±0.28	0.07
eGFR (mL/min/1.73m^2^)	85.37±19.02	86.24±17.66	-0.05
ACR (mg/g Cr)	33.73±170.65	25.65±134.45	0.05
Uric acid (mg/dL)	5.67±1.39	5.70±1.48	-0.02
Ankle-brachial index	1.04±0.10	1.04±0.09	0.00
baPWV	1639.41±448.62	1644.47±439.43	-0.01
***History of disease***			
Hypertension	385 (26.01)	203 (27.40)	-0.03
Diabetes Mellitus	125 (8.45)	80 (10.80)	-0.08
Heart disease	208 (14.05)	97 (13.09)	0.03
Stroke	64 (4.32)	18 (2.43)	0.10
Hyperlipidemia	192 (12.97)	108 (14.57)	-0.05
Gout	137 (9.26)	57 (7.69)	0.06
Cancer	46 (3.11)	24 (3.24)	-0.01
***Medication use***			
Hypertension medications	293 (19.8)	156 (21.05)	-0.03
Anti-diabetes medications	94 (6.35)	65 (8.77)	-0.09
Heart medications	128 (8.65)	58 (7.83)	0.03
Stroke medications	46 (3.11)	15 (2.02)	0.07
Hyperlipidemia medications	71 (4.80)	45 (6.07)	-0.06
***Outcome***			
All-cause mortality	179 (12.09)	83 (11.20)	0.03
Expanded CVD mortality	52 (3.51)	26 (3.51)	0.00

†: SD = standard deviation; hsCRP: high sensitivity C-Reactive Protein; eGFR: estimated Glomerular filtration rate; ACR: albumin-to-creatinine ratio; baPWV: brachial-ankle Pulse Wave Velocity.

Table 2 shows the univariate and multivariate analyses of the significant factors related to all-cause and expanded CVD mortality. In the multivariate Cox analysis, the significant factors of all-cause mortality were age (hazard ratio [HR]:1.10, 95% confidence intervals [CIs]:1.08–1.12), men (1.79, 1.24–2.60), single or widow (1.70, 1.20–2.41), smoking (1.84, 1.28–2.67), no leisure-time physical activity (1.61, 1.16–2.25), eGFR<30 (mL/min/1.73m^2^) (3.26, 1.55–6.87), and ACR≥300 (mg/g Cr) (4.44, 2.54–7.77). Significant factors for expanded CVD mortality were age (1.12, 1.09–1.16), BMI <18.5 (kg/m^2^) (6.27, 2.47–15.81), heart disease plus heart disease medication use (2.57, 1.38–4.76), stroke plus medication use (2.45, 1.15–5.23), and ABI≤0.9 (mm) (2.66, 1.23–5.76). Sex wasn’t significant but we entered it into final model due because sex was an important factor in literature [34]. The final risk scores based on the parameter estimates of regression coefficients are represented in Table 3. The risk predicting scores for all-cause mortality consisted of seven variables from 37 possible variables with the total score ranging from 0 to 18. The expanded CVD mortality risk score set consisted of six variables with a possible total score of 0–19. The predicted mortalities from all-causes were 0.1% to 99.9%, 0.3% to 100.0%, and 0.7% to 100.0% in the 3-, 5-, and 10-year periods, respectively. The mortalities from the expanded CVD were 0.1% to 100.0%, 0.1% to 100.0%, and 0.3% to 100.0% in the 3-, 5-, and 10-year periods, respectively, as shown in S2 Table.

**Table 2:** Cox model measured hazard ratio and 95% confidence intervals of all-cause and expanded CVD mortality.

	HR (95% CI)			
	All-cause mortality		Expanded CVD mortality	
Variables	Age-adjusted	Multivariate-adjusted	Age-adjusted	Multivariate-adjusted
***Socio-demographic factors***				
Age (years)	1.11 (1.10, 1.13)***	1.10 (1.08, 1.12)***	1.15 (1.12, 1.18)***	1.12 (1.09, 1.16)***
Sex				
Female	1.00	1.00	1.00	1.00
Male	1.53 (1.09, 2.14)*	1.79 (1.24, 2.60)**	1.74 (0.89, 3.37)*	1.93 (0.99, 3.74)
Marital status				
Married	1.00	1.00	1.00	
Single, widow	1.42 (1.02, 1.98)*	1.70 (1.20, 2.41)**	1.10 (0.59, 2.07)	
BMI (kg/m^2^)				
<18.5	1.96 (1.05, 3.65)*		4.06 (1.66, 9.93)**	6.27 (2.49, 15.81)***
18.5–24.9	1.00		1.00	1.00
25–29.9	1.09 (0.79, 1.50)		1.52 (0.83, 2.80)	1.38 (0.75, 2.57)
≥30	0.88 (0.43, 1.82)		1.46 (0.44, 4.85)	1.41 (0.42, 4.74)
***Lifestyle behaviors***				
Smoking				
No	1.00	1.00	1.00	
Yes	2.43 (1.72, 3.44)***	1.84 (1.28, 2.67)**	1.73 (0.84, 3.56)	
Physical activity				
Yes	1.00	1.00	1.00	
No	1.77 (1.29, 2.44)***	1.61 (1.16, 2.25)**	1.98 (1.11, 3.56)*	
***Biomarker***†				
Blood pressure (mmHg)				
SBP<120 and DBP<80	1.00		1.00	
SBP≥120 or DBP≥80	0.84 (0.52, 1.35)		2.21 (0.53, 9.29)	
Total cholesterol (mg/dL)				
<200	1.00		1.00	
≥200	0.89 (0.66, 1.20)		0.84 (0.48, 1.47)	
Triglyceride (mg/dL)				
<150	1.00		1.00	
≥150	1.19 (0.85, 1.66)		0.90 (0.46, 1.74)	
HDL (mg/dL)				
Male: >40; female:>50	1.00		1.00	
Male: ≤40; female: ≤50	0.92 (0.68, 1.23)		1.25 (0.72, 2.18)	
LDL (mg/dL)				
<110	1.00		1.00	
≥110	0.64 (0.46, 0.90)*		0.85 (0.44, 1.65)	
eGFR (mL/min/1.73m^2^)				
≥60	1.00	1.00	1.00	
30–59	1.75 (1.22, 2.50)**	1.39 (0.97, 2.01)	1.72 (0.92, 3.22)	
<30	5.37 (2.63, 10.95)***	3.26 (1.55, 6.87)**	1.70 (0.22, 13.05)	
ACR (mg/g Cr)				
<30	1.00	1.00	1.00	
30–299	1.64 (1.16, 2.31)**	1.42 (0.99, 2.03)	1.40 (0.73, 2.68)	
≥300	6.27 (3.7, 10.63)***	4.44 (2.54, 7.77)***	9.12 (3.75, 22.19)***	
***History of disease***				
Hypertension				
No	1.00		1.00	
Yes				
Hypertension medications				
No	1.05 (0.59, 1.87)		2.76 (1.17, 6.53)*	
Yes	1.04 (0.76, 1.44)		1.79 (0.99, 3.26)	
Diabetes Mellitus				
No	1.00		1.00	
Yes				
DM medications				
No	1.22 (0.57, 2.61)		1.11 (0.27, 4.61)	
Yes	1.48 (1.00, 2.21)		1.41 (0.68, 2.91)	
Heart disease				
No	1.00		1.00	1.00
Yes				
Heart medications				
No	1.36 (0.79, 2.33)		1.15 (0.35, 3.81)	1.59 (0.47, 5.32)
Yes	1.31 (0.91, 1.89)		2.70 (1.49, 4.90)**	2.57 (1.38, 4.76)**
Stroke				
No	1.00		1.00	1.00
Stroke medications				
No	1.51 (0.62, 3.68)		1.10 (0.15, 7.99)	1.33 (0.18, 9.93)
Yes	1.49 (0.90, 2.46)		2.75 (1.34, 5.65)**	2.45 (1.15, 5.23)*
Ankle-brachial index				
>0.9	1.00		1.00	1.00
≤0.9	1.61 (0.98, 2.62)		2.55 (1.19, 5.48)*	2.66 (1.23, 5.76)*

HR: hazard ratio; CI: confidence interval; SBP: systolic blood pressure; DBP: diastolic blood pressure; HDL: high-density lipoprotein; LDL: low-density lipoprotein; eGFR: estimated Glomerular filtration rate; ACR: albumin-to-creatinine ratio.

*:p<0.05

**:p<0.01

***:p<0.001.

**Table 3:** Parameter estimates of regression coefficient and risk score of predictors for all-cause and expanded CVD mortality from the final multivariate Cox’s proportional hazards model.

	All-cause mortality			Expanded CVD mortality		
Variables	β (SE)	p-value	Risk score	β (SE)	p-value	Risk score
***Socio-demographic factors***						
Age (years)	0.09 (0.01)	<0.001	0–9	0.11 (0.02)	<0.001	0–9
Sex (male)	0.58 (0.19)	0.002	1	0.66 (0.34)	0.05	1
Single, widow	0.53 (0.18)	0.003	1			
BMI (kg/m^2^) (ref: 18.5–24.9)						
<18.5				1.84 (0.47)	<0.001	3
25–29.9				0.32 (0.32)	0.30	1
≥30				0.34 (0.62)	0.58	1
***Lifestyle behaviors***						
Smoking (ref: No)	0.61 (0.19)	0.001	1			
Physical activity (ref: Yes)	0.48 (0.17)	0.005	1			
***Biomarker*†**						
eGFR (mL/min/1.73m^2^) (ref: ≥60)						
30–59	0.33 (0.19)	0.08	1			
<30	1.18 (0.38)	0.002	2			
ACR (mg/g Cr) (ref: <30)						
30–299	0.35 (0.18)	0.06	1			
≥300	1.49 (0.29)	<0.001	3			
***History of disease***						
Heart disease (ref: No)						
Heart medications						
No				0.29 (1.03)	0.78	1
Yes				0.90 (0.39)	0.02	2
Stroke (ref: No)						
Stroke medications						
No				0.46 (0.62)	0.46	1
Yes				0.94 (0.32)	0.003	2
Ankle-brachial index						
≤0.9				0.98 (0.39)	0.01	2

β: parameter estimate; SE: standard error; eGFR: estimated Glomerular filtration rate; ACR: albumin-to-creatinine ratio.

The areas under receiver operating curves (AUCs) and their 95% CIs for all-cause mortality were 0.97 (0.94–0.99), 0.96 (0.94–0.98), and 0.88 (0.83–0.93) within the 3-, 5-, and 10-year periods, respectively. The AUCs for expanded CVD mortality were 0.97 (0.91–1.00), 0.98 (0.95–1.00), and 0.84 (0.75–0.93), respectively (S1 Fig). The similar results of AUCs were found by using time-dependent ROC curve analysis. They were 0.97, 0.96, and 0.90 for all-cause mortality, and 0.98, 0.98, and 0.88 for expanded CVD mortality in the 3-, 5-, and 10-year periods, respectively. In the validation set, the fitness indices for two mortality models exhibited excellent performance in the 3- and 5-year periods (AUC≥0.9). The results for comparing predicted deaths with observed deaths in the validation sets were similar using the Hosmer–Lemeshow test (all P>0.3) (S2 Fig). The 5000 bootstrap sampling was used to assess the internal validity of the mortality models and to estimate its performance in the validation set. The intercept and slope values were −0.01 and 0.94 for the calibration of the 10-year all-cause mortality, respectively, and 0.01 and 0.22 for the 10-year expanded CVD mortality, respectively. This result indicated that our prediction model had good prediction for 10-year all-cause mortality, but underestimated the 10-year CVD mortality.

**Fig 1:**
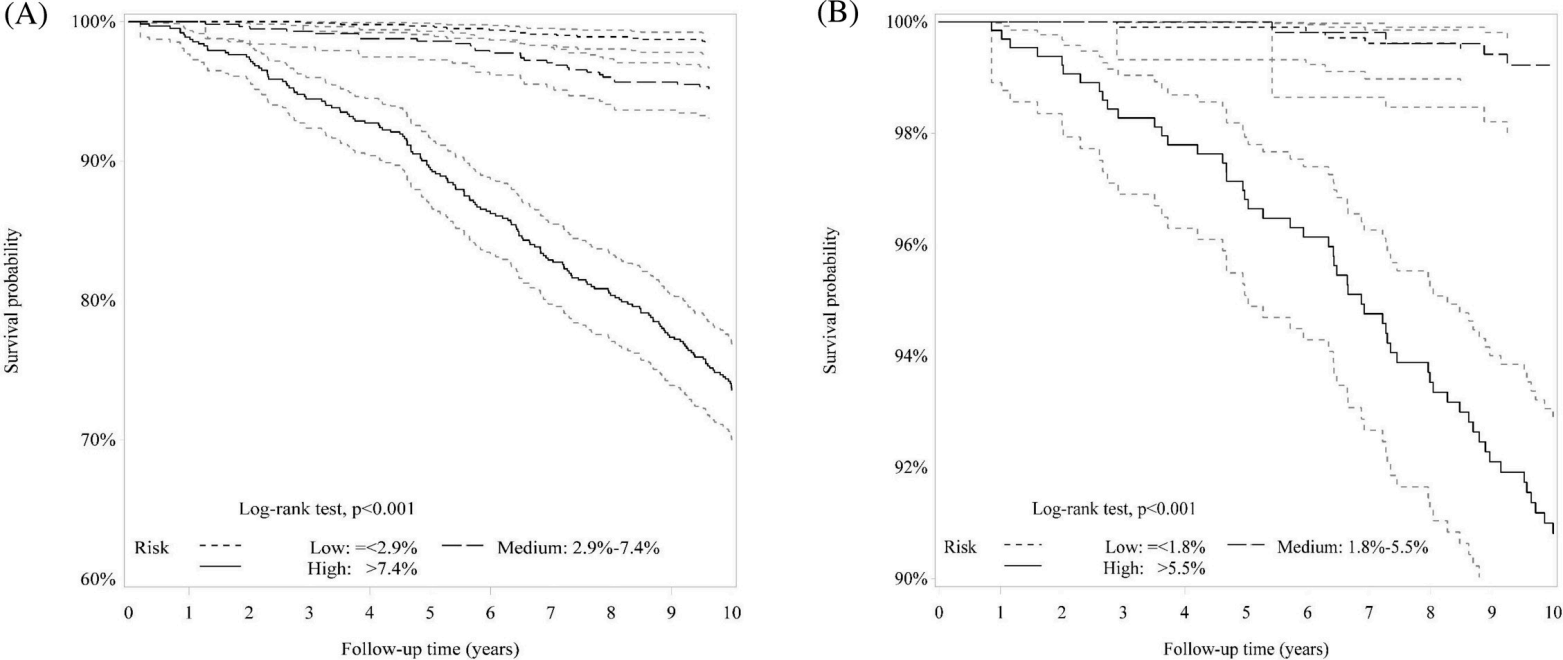
Kaplan-Meier survival curves for (A) all-cause and (B) expanded CVD mortality according to the low, medium, and high-risk groups.

As shown in Fig 1, the Kaplan–Meier survival curves for all-cause and expanded CVD mortality were plotted by the different tertile risk groups (all log-rank test, P<0.001). Compared with the low-risk group, the medium and high-risk groups had HR of 2.43 (95% CI 1.41–4.16) and 17.53 (11.39–26.98) for all-cause mortality and 2.03 (0.59–7.00) and 26.26 (10.58–65.15) for expanded CVD mortality, respectively.

## Discussion

Our study developed novel prediction models consisting of renal function and arterial stiffness for all-cause mortality and expanded CVD mortality among middle-aged and elderly community-dwelling adults in Taiwan. These two mortality prediction models indicated excellent discrimination and good calibration for all-cause mortality in the validation sets. In addition, the two models are parsimony and reliable in computing an individual’s risks of overall mortality and expanded cardiovascular mortality based on the traditional risk factors and novel biomarkers of atherosclerosis. In addition, the strength of the present study is to provide a relatively long-term 10-year all-cause and expanded-cardiovascular mortality model in the form of risk-point system.

The all-cause mortality prediction models have been developed in previous studies [9–21]. However, these models reported lower C-values compared with those in our models. Our findings were consistent with some previous studies stating that both eGFR and proteinuria are key predictors for the all-cause mortality in general Chinese population [35, 36]. These two renal measures have contributed to their independent effect on mortality [35, 36] and to the leading risk factors of premature death, such as diabetes, hypertension, dyslipidemia, and obesity [37–39]. This condition results in the high ability on explaining most cases of premature death. Similarly, our predictive model also revealed that strong, independent, and graded associations were observed between all-cause mortality and two important renal function-related markers, namely, decreased GFR and increased urinary albumin excretion. These two variables were not considered simultaneously in the general population in the previous models [9–21].

ABI measurement is recognized as a simple to perform, accurate, noninvasive, and inexpensive test; the test of ABI≤0.90 (mm) is a sensitive index to detect either symptomatic or asymptomatic peripheral arterial disease [40]. Notably, asymptomatic PAD is prevalent in 5.4% among Taiwanese individuals [41]. One systematic review described that low ABI has increased by more than three-fold risk of CVD death and also identified individuals at high risk of CVD mortality to allow for early interventions [23]. Our expanded CVD mortality prediction model included the generally accepted CVD risk factors and a novel predictor of ABI to improve the predictive ability of the models. However, the well-established risk factors such as cholesterol and hypertension, included to predict 10-year CVD mortality in the Framingham Score, are not included in our model [42]. The possible reason for this difference may be our study considered baPWV and histories of heart disease and stroke that may explain the effect of cholesterol and hypertension.

The expanded cardiovascular diseases mortality was a composite measure of cardiovascular-related mortality, i.e. measurements based on multiple items for cause of death that are cardiovascular-related. CKD has become recognized as a key independent risk factor for cardiovascular disease (CVD) [43]. It is now increasingly apparent that individuals with CKD are more likely to die from CVD than to develop end stage renal disease *(*ESRD). Thus, the death of cause due to renal diseases has been considered as CVD-related death. The advantage of a composite measure is that it typically results in an increase in the incidence rates of the composite endpoint, thus it increases its statistical power. Many prior studies had adopted the expanded cardiovascular diseases mortality as one of their outcome measures [39, 44–49]. In prior studies reporting prediction models in general population, only two studies provided risk function for CVD mortality [13, 19]. One had been conducted in Western population focusing on dietary quality score [13] for predicting 10-year CVD mortality with a highest c-statistic value of 0.805, and the other had been conducted in Chinese population focusing on frailty markers for predicting 4-year CVD mortality with a c-statistic value of 0.80 [19]. Our study provided the risk score for expand CVD mortality focusing on traditional CVD risk factors along with renal function and arterial stiffness with good discriminatory power reaching as high as 0.98, 0.98, and 0.88 in the 3-, 5-, and 10-year periods.

Mortality predictive models have important implications for human and healthcare resource management. Therefore, we improved the previous Taiwanese mortality prediction models [18–21]. We also developed two novel models for all-cause and expanded CVD mortality by involving integrating new and novel biomarkers, considering long-term risk evaluation, and validating and calibrating multivariate risk models. Bootstrap sampling approach was further used to assess the internal validity of the present models based on 5000 samples. Our results showed good calibration for the present model of all-cause mortality with a mean slope close to 1.0.

Our study has several advantages. First, this study included one well-defined community-based cohort of middle-aged and elderly adults and the inclusion of powerful predictors of renal function and ABI. Second, the recruitment process followed built-in standardized protocols and instruments to ensure the high-quality of data collection. Our study has six limitations. First, the effects of time-varying predictors are not considered. Second, external validation was not performed because no external sample was available. Third, our study only considered expanded CVD mortality because TCHS was designed to explore the issues about cardiovascular risk factors. Other cause-specific mortalities were not considered because our study lacks the factors associated with other causes of death. Fourth, our study has a relatively small sample size compared with previous studies [10–12, 16, 21]. In addition, the calibration results indicated that our prediction for 10-year expanded CVD mortality was underestimated due to the small sample size although it has good predictive ability. Fifth, the generalization of this prediction model may vary with the medical care system. Our study was based on a population under a universal national health care system. Such a selection bias may limit our findings to be generalized to other medical care systems, but they may be generalizable to other populations under similar medical care system. Finally, the study subjects of derivation and validation sets are both of Chinese. Thus, our study findings may not be generalized to other ethnic ancestries.

In conclusion, using Taiwan community survey and survival data, we have created two simple and applicable prediction score systems with excellent calibration and high discrimination and compared these systems with other existing models. These two risk score systems exhibit good ability to predict all-cause and expanded cardiovascular mortality risk factors in the general community population and reveal the relative importance of predictors. For individuals, hospitals, or healthcare providers, these systems could be used to obtain the estimates of 3-, 5-, and 10-year probabilities of death for planning lifestyle intervention or treatment on mortality reduction or patients’ quality of life improvement.

## Supporting information

S1 Fig Receiver operating characteristic curve (ROC) for (a) 3-year (b) 5-year (c) 10-year all-cause mortality and for (d) 3-year (e) 5-year (f) 10-year expanded CVD mortality in validation set.
(DOCX)

S2 Fig Predicted versus observed death numbers according to deciles of (a) 3-year (b) 5-year (c) 10-year all-cause mortality and (d) 3-year (e) 5-year (f) 10-year expanded CVD mortality in validation set.
(DOCX)

S1 Table The reasons for the ineligible persons. (DOCX)

S2 Table 2. 3-, 5- and 10-year estimated risk for all-cause and expanded CVD mortality of each possible sum of points. (DOCX)
