# Supplementary material for: Derivation and validation of 10-year all-cause and cardiovascular disease mortality prediction model for middle-aged and elderly community-dwelling adults in Taiwan
Source: PLoS One. 2020 Sep 14;15(9):e0239063. doi: 10.1371/journal.pone.0239063 (PMC7489508; doi:10.1371/journal.pone.0239063)
Supplement: S2 Fig — Predicted versus observed death numbers according to deciles of (a) 3-year (b) 5-year (c) 10-year all-cause mortality and (d) 3-year (e) 5-year (f) 10-year expanded CVD mortality in validation set. (DOCX) [file pone.0239063.s002.docx]

|  |  |  |
| --- | --- | --- |
| (d) | (e) | (f) |

**Supplemental figure 2.** Predicted versus observed death numbers according to deciles of (a) 3-year (b) 5-year (c) 10-year all-cause mortality and (d) 3-year (e) 5-year (f) 10-year expanded CVD mortality in validation set
