# Supplementary material for: Derivation and validation of 10-year all-cause and cardiovascular disease mortality prediction model for middle-aged and elderly community-dwelling adults in Taiwan
Source: PLoS One. 2020 Sep 14;15(9):e0239063. doi: 10.1371/journal.pone.0239063 (PMC7489508; doi:10.1371/journal.pone.0239063)
Supplement: S1 Table — (DOCX) [file pone.0239063.s003.docx]

**Supplemental table 1**. The reasons for the ineligible persons

| Reasons | *n* (%) |
| --- | --- |
| Death | 18 (2.40%) |
| Hospitalization or imprisonment | 14 (1.87%) |
| Living abroad | 39 (5.20%) |
| Moving out of the area | 411 (54.80%) |
| Living in their children's home | 7 (0.93%) |
| Sampling frame mistakes | 59 (7.87%) |
| Not being at home during 3 visits made by interviewers | 202 (26.93%) |
| Total | 750 |
