# Supplementary material for: Derivation and validation of 10-year all-cause and cardiovascular disease mortality prediction model for middle-aged and elderly community-dwelling adults in Taiwan
Source: PLoS One. 2020 Sep 14;15(9):e0239063. doi: 10.1371/journal.pone.0239063 (PMC7489508; doi:10.1371/journal.pone.0239063)
Supplement: S2 Table — (DOCX) [file pone.0239063.s004.docx]

**Supplemental table 2**. 3-, 5- and 10-year estimated risk for all-cause and expanded CVD mortality of each possible sum of points

| Point total | Predicted risk of all-cause mortality (%) | | | Predicted risk of expanded CVD mortality (%) | | |
| --- | --- | --- | --- | --- | --- | --- |
|  | 3-year risk | 5-year risk | 10-year risk | 3-year risk | 5-year risk | 10-year risk |
| 0 | 0.1% | 0.3% | 0.7% | 0.1% | 0.1% | 0.3% |
| 1 | 0.2% | 0.5% | 1.2% | 0.1% | 0.2% | 0.6% |
| 2 | 0.4% | 0.7% | 1.8% | 0.2% | 0.3% | 1.0% |
| 3 | 0.6% | 1.2% | 2.9% | 0.3% | 0.6% | 1.8% |
| 4 | 0.9% | 1.9% | 4.7% | 0.6% | 1.0% | 3.1% |
| 5 | 1.5% | 3.0% | 7.4% | 1.1% | 1.8% | 5.5% |
| 6 | 2.4% | 4.8% | 11.6% | 1.9% | 3.2% | 9.5% |
| 7 | 3.8% | 7.6% | 18.0% | 3.4% | 5.6% | 16.2% |
| 8 | 6.0% | 11.8% | 27.2% | 5.9% | 9.6% | 26.9% |
| 9 | 9.5% | 18.3% | 39.9% | 10.2% | 16.4% | 42.5% |
| 10 | 14.8% | 27.7% | 55.9% | 17.3% | 27.2% | 62.5% |
| 11 | 22.6% | 40.6% | 73.1% | 28.6% | 43.0% | 82.4% |
| 12 | 33.7% | 56.6% | 87.8% | 44.9% | 63.1% | 95.4% |
| 13 | 48.3% | 73.8% | 96.6% | 65.3% | 82.9% | 99.6% |
| 14 | 65.3% | 88.3% | 99.6% | 84.6% | 95.6% | 100.0% |
| 15 | 81.7% | 96.8% | 100.0% | 96.4% | 99.6% | 100.0% |
| 16 | 93.4% | 99.6% | 100.0% | 99.7% | 100.0% | 100.0% |
| 17 | 98.7% | 100.0% | 100.0% | 100.0% | 100.0% | 100.0% |
| 18 | 99.9% | 100.0% | 100.0% | 100.0% | 100.0% | 100.0% |
| 19 |  |  |  | 100.0% | 100.0% | 100.0% |
